# Association between degree of exposure to the Hospital Value Based Purchasing Program and 30-day mortality: experience from the first four years of Medicare’s pay-for-performance program

**DOI:** 10.1186/s12913-019-4562-7

**Published:** 2019-12-02

**Authors:** Souvik Banerjee, Danny McCormick, Michael K. Paasche-Orlow, Meng-Yun Lin, Amresh D. Hanchate

**Affiliations:** 1 Disparities Research Unit and The Mongan Institute, Massachusetts General Hospital, Boston, MA USA; 2 Harvard Medical School, Boston, USA; 3 Cambridge Health Alliance, Cambridge, MA USA; 4 Section of General Internal Medicine, Boston University School of Medicine, 801 Massachusetts Ave #2092, Boston, MA 02118 USA; 5 VA Boston Healthcare System, Boston, MA USA

**Keywords:** Hospital performance, Pay for performance, Hospital value based purchasing program, 30-day mortality

## Abstract

**Background:**

The Hospital Value Based Purchasing Program (HVBP) in the United States, announced in 2010 and implemented since 2013 by the Centers for Medicare and Medicaid Services (CMS), introduced payment penalties and bonuses based on hospital performance on patient 30-day mortality and other indicators. Evidence on the impact of this program is limited and reliant on the choice of program-exempt hospitals as controls. As program-exempt hospitals may have systematic differences with program-participating hospitals, in this study we used an alternative approach wherein program-participating hospitals are stratified by their financial exposure to penalty, and examined changes in hospital performance on 30-day mortality between hospitals with high vs. low financial exposure to penalty.

**Methods:**

Our study examined all hospitals reimbursed through the Medicare Inpatient Prospective Payment System (IPPS) – which include most community and tertiary acute care hospitals – from 2009 to 2016. A hospital’s financial exposure to HVBP penalties was measured by the share of its annual aggregate inpatient days provided to Medicare patients (“Medicare bed share”). The main outcome measures were annual hospital-level 30-day risk-adjusted mortality rates for acute myocardial infarction (AMI), heart failure (HF) and pneumonia patients. Using difference-in-differences models we estimated the change in the outcomes in high vs. low Medicare bed share hospitals following HVBP.

**Results:**

In the study cohort of 1902 US hospitals, average Medicare bed share was 61 and 41% in high (*n* = 540) and low (*n* = 1362) Medicare bed share hospitals, respectively. High Medicare bed share hospitals were more likely to have smaller bed size and less likely to be teaching hospitals, but ownership type was similar among both Medicare bed share groups.. Among low Medicare bed share (control) hospitals, baseline (pre-HVBP) 30-day mortality was 16.0% (AMI), 10.9% (HF) and 11.4% (pneumonia). In both high and low Medicare bed share hospitals 30-day mortality experienced a secular decrease for AMI, increase for HF and pneumonia; differences in the pre-post change between the two hospital groups were small (< 0.12%) and not significant across all three conditions.

**Conclusions:**

HVBP was not associated with a meaningful change in 30-day mortality across hospitals with differential exposure to the program penalty.

## Background

The three aims of better health, better care, and lower costs of the U.S. Department of Health and Human Services’ National Quality Strategy are at the focal-point of the Centers for Medicare and Medicaid Services’ (CMS’) notion of value [1]. The Affordable Care Act (ACA), passed in 2010, introduced hospital-based reforms that sought to move from volume-based to value-based care through financial incentives to hospitals to improve performance [2]. The Hospital Value Based Purchasing Program (HVBP) is a key mechanism through which CMS ties financial payment adjustments to hospital performance for acute care hospitals paid under the Inpatient Prospective Payment System (IPPS), covering most community and tertiary acute care hospitals in the US [3]. Starting in fiscal year (FY) 2013, IPPS hospitals were subject to a financial penalty or bonus – in the form of a percentage reduction or increase not just in the reimbursement for these admission cohorts but for all admissions of Medicare patients – depending on their past performance along multiple domains of inpatient care: patient experience; 30-day mortality; clinical process of care; and efficiency [4]. The 30-day mortality measure, accounting for 25% of the total score, was introduced into the HVBP performance scoring in 2014, and applied to acute myocardial infarction (AMI), heart failure (HF) and pneumonia admission cohorts. Maximum penalty was 1% (reduction in Medicare reimbursement) in 2013 and increased to 1.75% in 2016 and 2% since 2017.

Evidence on the impact of HVBP on the target outcomes is limited. Two prior studies, which found that HVBP was not associated with any change in 30-day mortality, compared mortality changes in IPPS hospitals with those in non-IPPS hospitals [5, 6]. These studies used critical access hospitals or hospitals in the state of Maryland as the comparison group because neither were subject to the HVBP penalties. However, critical access hospitals are small rural hospitals (with fewer than 25 inpatient beds) whose operations may be structurally different from IPPS hospitals [7]. Furthermore, as these hospitals are not mandated to report their performance, those who voluntarily report performance may be selectively different from those that do not. Similarly, while hospitals in the state of Maryland were not exposed to the HVBP, as they are not paid under IPPS, they were exposed to a similar set of state imposed financial incentives to improve quality during the relevant time period [8, 9]. Moreover, these studies had limited follow-up data – one to two years – after the start of the HVBP and were unable to assess longer-term changes in the mortality outcomes. In this study, we focused only on IPPS hospitals and compared the performance of hospitals in which Medicare patients account for a high share of bed days with hospitals in which Medicare patients account for a low share of bed days using 4 years of follow-up data on mortality since the start of the HVBP. Our rationale, based on prior work on the impact of pay-for-performance programs, is that the incentives from the HVBP payment adjustments will have a larger impact on hospitals that rely on Medicare patients to a greater extent [6, 10]. Hospitals with lower reliance on Medicare patients (“low Medicare share hospitals”) will effectively experience a lower “dose” of the incentive compared to those with higher reliance (“high Medicare share hospitals”).

Using publicly reported data on hospital performance from 2009 to 2016, we examined the association between HVBP incentive size and changes in 30-day mortality by comparing pre- to post-HVBP changes in hospital 30-day mortality among high Medicare share hospitals with those of low Medicare share hospitals. We examined 30-day mortality changes for the three admission cohorts (AMI, HF and pneumonia) that were all introduced into the HVBP in 2014.

## Methods

### Data sources and sample

The data for this study comes from two publicly available sources: CMS Hospital Compare [11] (2009–2016) and CMS Final Impact Rule [12] (2009–2016) as well as from the American Hospital Association Annual Survey [13] (2009) and Census Bureau’s American Community Survey [14]. The study sample comprised all IPPS hospitals from 2009 to 2016 (2756 IPPS hospitals in 2009 and 2607 IPPS hospitals in 2016); CAHs, hospitals in the State of Maryland, pediatric hospitals, long term care facilities, psychiatric hospitals, rehabilitation hospitals, and Veterans Affairs hospitals were excluded [4]. Additionally, hospitals were excluded if their IPPS status changed during the study period or if publicly reported 30-day risk-adjusted morality was unavailable for any study year. Therefore, all included hospitals have balanced data for all study years (2009–2016).

### Outcomes, independent variable and hospital characteristics

Our outcomes were hospital-level 30-day risk adjusted mortality rates for AMI, HF, and pneumonia reported annually by the CMS Hospital Compare program [15]; these refer to mortality experienced by Medicare patients admitted to a hospital. Our analytic data consisted of annual longitudinal observations for each of these three diagnoses at IPPS hospitals. According to the Hospital Compare program, for each hospital, the risk adjusted rate of 30-day mortality reported each year for each cohort is based on eligible admissions during that year and in the preceding 2 years, and adjusted for compositional differences across hospitals in patient age, sex, comorbid health conditions and other unobserved, but systematic, hospital effects [16]. For the pneumonia cohort, we did not include 2016 performance since CMS’ identification criteria for pneumonia admissions was modified in 2016 to include admissions with aspiration pneumonia as a principal discharge diagnosis, and admissions with sepsis as a principal discharge diagnosis that have a secondary diagnosis of pneumonia [17].

Our identification of high vs. low Medicare share hospitals was based on the share of aggregate inpatient days of care provided to Medicare-reimbursed patients out of total inpatient days for all patients in 2009 (baseline pre-HVBP year). Specifically, we defined a dichotomous indicator of high Medicare share hospitals that groups hospitals with Medicare share inpatient days greater than or equal to 55% as high Medicare share hospitals and the other hospitals as low Medicare share hospitals. Due to a dearth of prior work or theoretical guidelines in grouping hospitals by Medicare share, we also examined different categorizations including with different cut-offs and examined the sensitivity of study findings to these alternative cut-offs.. [18, 19]. Due to the similarity of findings across the different specifications, we have reported findings based on the 55% cut-off as our preferred estimate; estimates from alternative cut-offs (50 and 60%) are reported in the Appendix.

The hospital characteristics included in our analysis as regression covariates were: bed size (less than 100, 100–199, and 200 or more); teaching hospital status - a binary variable indicating membership in the Council of Teaching Hospitals; ownership (not-for-profit, government non-federal, and for-profit); and region (Northeast, Midwest, South, West). In addition, we also included time-varying characteristics: hospital average daily patient census; proportion of low income patients at hospital level (disproportionate share hospital [DSH] %); state-level annual poverty rate; state-level annual unemployment rate.

### Statistical analysis

**Fig. 1:**
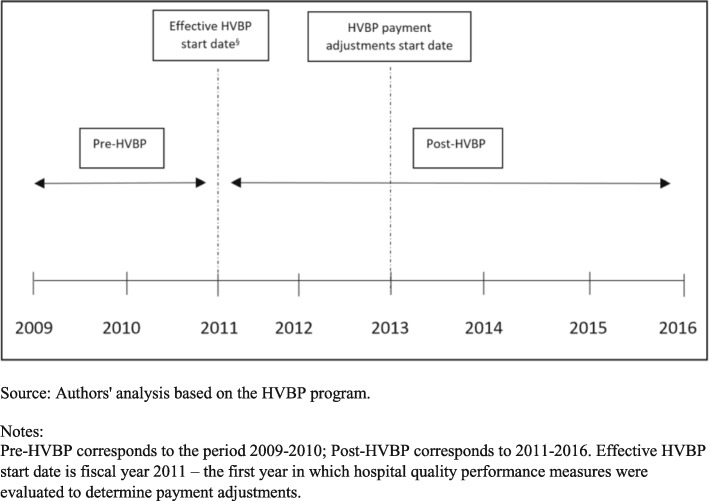
Timeline of analysis window

We compared the hospital characteristics of the high vs. low Medicare share hospitals in the baseline year 2009. We plotted annual average mortality rate for each condition from 2009 to 2016 for high and low Medicare share hospitals; compared the baseline difference in mortality rate between high and low Medicare share hospitals for each condition; and estimated linear time series models to capture average change in annual mortality rates over the study period for the targeted conditions among the two groups of hospitals. We evaluated the association of the HVBP incentive size with the mortality outcomes using a difference-in-differences type approach, whereby pre- vs. post-HVBP changes in the outcome in high Medicare share hospitals were contrasted with corresponding changes in low Medicare share hospitals. In identifying pre- and post-HVBP periods, we note that while the HVBP was announced in March 2010, with passage of the ACA, the 30-day mortality measures for AMI, HF, and pneumonia were introduced in FY 2014. However, hospital performance on 30-day mortality was based on all eligible hospitalizations during the prior 3 year period, and for the first program year (FY 2014) hospital performance was based on discharges during July 1, 2011 – June 30, 2013 [15]. Since this evaluation plan was announced in 2010 soon after the passage of the ACA, we consider the start date of the HVBP to be 2011, the first year in which patient outcome measures were evaluated to determine payment adjustments for FY 2014. We defined a dichotomous measure of the post-HVBP period that was equal to 1 for the time period 2011–2016 (post-HVBP) and 0 for observations between 2009 and 2010 (pre-HVBP) (Fig. 1). As post-HVBP period is longer than the pre-HVBP period, in sensitivity analyses we examined if use of a shorter post-HVBP period (2011–2013 and 2014–2016 separately) affects the main findings; these analyses also examine if the results differ if we used earlier (2011–2013) and later (2014–2016) post periods. To examine for heterogeneity in mortality change we used a three-way difference-in-differences regression model specification and estimated mortality change estimates for hospitals grouped by teaching status, ownership and bed size.

For our main analysis, to estimate the association between hospital mortality performance and the degree of exposure to the HVBP, we used linear regression models with difference-in-differences specifications that included indicators for high Medicare share hospitals and post-HVBP time period, and for the interaction (product) of high Medicare share hospitals and post-HVBP. We also included as covariates, indicators of the aforementioned hospital characteristics measured in the baseline year (2009). The coefficient estimate of the interaction term gives the excess pre- to post-HVBP change in mortality rate for high Medicare share hospitals compared to that for low Medicare share hospitals [20, 21]. As the difference-in-differences specification assumes similarity in pre-HVBP longitudinal changes in each mortality measure between high vs. low Medicare share hospitals, we tested the validity of this assumption (“parallel trends test”) using alternative difference-in-differences models using only pre-HVBP data [22]. The results are reported in Table 3 in Appendix. We used a linear (hospital-level) random effects regression model with heteroscedasticity-robust standard errors [23, 24]. In sensitivity analysis with respect to the random effects specification, we also performed similar estimation using a fixed effects specification to control for systematic time-invariant unobserved hospital characteristics [24]. For each model, we included year fixed effects to adjust for secular trends in the mortality outcomes.

Given potential changes in 30-day readmission rate for AMI, HF, and pneumonia associated with the Hospital Readmission Reduction Program (HRRP), another CMS incentive program that was introduced alongside the HVBP in 2010, there may be unintended spillover effects (of the HRRP) on 30-day mortality rates for the aforementioned conditions [25]. As a sensitivity analysis, we re-estimated variants of our main models that included 30-day risk adjusted readmission rate for the corresponding admission cohort as an additional covariate; a significant difference between the difference-in-differences estimates in the models without and with adjustment for the readmission rate would indicate possible spillover effects.

All statistical analyses in this study were performed using Stata version 14.1 [26]. The Institutional Review Board of the Boston University School of Medicine considered this study exempt from human subjects review as no person-level data was involved.

## Results

The final study sample comprised 1902 hospitals in each year between 2009 and 2016. We compared the characteristics of the high Medicare share hospitals vs. the low Medicare share hospitals in 2009 (Table 1). The mean share of Medicare inpatient days was 61% for high Medicare share hospitals and 41% for low Medicare share hospitals. A significantly lower proportion of high Medicare share hospitals compared to low Medicare share hospitals were teaching hospitals (1.1% vs. 17.2%). Hospital ownership types were similar across both hospital groups, but a larger proportion of the high Medicare share hospitals had smaller bed size (≤99 beds; 28.1% vs. 13.2%) and were in the South (45.2% vs. 34.3%).

**Table 1:** Hospital characteristics in baseline year (2009) for high vs. low Medicare share hospitals

	High Medicare share hospitals (*N* = 540)	Low Medicare share hospitals (*N* = 1362)	All hospitals (*N* = 1902)	*p* value^a^
	No. (Percent)	No. (Percent)	No. (Percent)	
Medicare bed share, Mean (Std. Dev)	0.61 (0.05)	0.41 (0.11)	0.46 (0.13)	< 0.001
Teaching hospital (COTH)^b^, n (%)	6 (1.1)	234 (17.2)	240 (12.6)	< 0.001
Ownership, n (%)				0.506
Not-for-profit	358 (66.3)	927 (68.1)	1285 (67.6)	
Govt. non-fed	72 (13.3)	189 (13.9)	261 (13.7)	
For-profit	110 (20.4)	246 (20.4)	356 (18.7)	
Bed size, n (%)				< 0.001
< 99	152 (28.1)	180 (13.2)	332 (17.5)	
100–199	176 (32.6)	354 (26.0)	530 (27.9)	
> =200	212 (39.3)	828 (60.8)	1040 (54.7)	
Region, n (%)				< 0.001
Northeast	105 (19.4)	267 (19.6)	372 (19.6)	
Midwest	163 (30.2)	293 (21.5)	456 (24.0)	
South	244 (45.2)	467 (34.3)	711 (37.4)	
West	28 (5.2)	335 (24.6)	363 (19.1)	
Average daily census, Mean (Std. Dev)	98.9 (87.7)	181.4 (161.8)	158.0 (149.4)	< 0.001
DSH %, Mean (Std. Dev)	31.7% (19.4%)	31.7% (19.4%)	28.3% (18.4%)	< 0.001
Poverty rate %, Mean (Std. Dev)	14.5% (2.7%)	14.4% (2.4%)	14.5% (2.5%)	0.463
Unemployment rate %, Mean (Std. Dev)	10.1% (1.9%)	9.9% (1.7%)	10.0% (1.7%)	0.136

Source: Authors’ analysis of American Hospital Association Annual Survey data (2009)

Hospitals that appear in any of the following samples are included in the table: *AMI* Acute myocardial infarction, *HF* Heart failure, and pneumonia

High Medicare share hospitals: Hospitals with Medicare share inpatient days > = 55%; low Medicare share hospitals: Hospitals with Medicare share inpatient days < 55%

^a^Difference between high and low Medicare share hospitals; t-test for continuous variables and chi-square test for categorical variables

^b^Member of Council of Teaching Hospital of the Association of American Medical Colleges

**Fig. 2:**
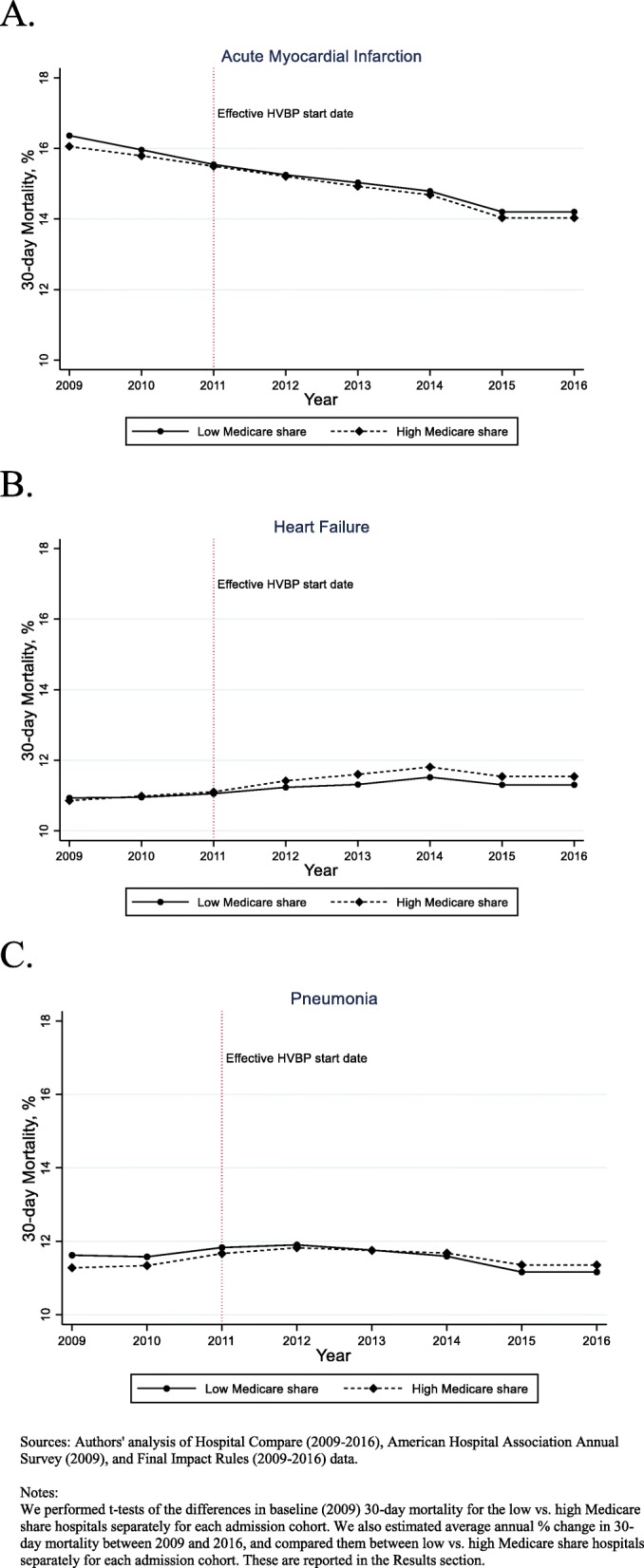
Trends in **a** acute myocardial infarction (AMI), **b** heart failure (HF), and **c** pneumonia 30-day risk adjusted mortality rates (2009–2016)

Figure 2 presents the longitudinal trends in the 30-day risk adjusted morality rate for AMI, HF, and pneumonia. The average annual change in mortality rate between 2009 and 2016 for low vs. high Medicare share hospitals for AMI was − 2.0%% vs. 2.04% [*p*-value for difference between the two groups = 0.53], for HF was 1.0% vs. 1.1% (*p*-value = 0.48) and for pneumonia was − 0.2% vs. 0.2% [*p*-value = 0.003].

The results of the association between a hospital’s degree of reliance on Medicare patients (and thus the effective incentive size under the HVBP) and 30-day risk adjusted mortality rate for AMI, HF, and pneumonia using the difference-in-differences analysis are presented in Table 2 (full regression estimates in Table 4 in Appendix). There was a secular decrease in 30-day AMI mortality rates between pre- and post-HVBP periods in both high and low Medicare share hospitals. Adjusted for trends and covariate differences, the difference in change in the high Medicare share hospitals was not significant (0.03, 95% confidence interval (CI) [− 0.13, 0.19%]). Similarly, for HF and pneumonia admissions, there was no significant difference in the changes in high vs. low Medicare share hospitals. We found that high patient census was associated with lower mortality across all three admission cohorts, while mortality was higher in government non-federal hospitals (for AMI and pneumonia admissions) and in the South.

**Table 2:** Difference in 30-day risk adjusted mortality trends between high and low Medicare share hospitals (2009–2016)

	Average 30-day risk-adjusted mortality	
	High Medicare share hospitals	Low Medicare share hospitals
A. Acute myocardial infarction		
Pre-HVBP: Mean (SD)	15.9% (1.7%)	16.0% (1.8%)
Post-HVBP: Mean SD)	14.8% (1.6%)	14.7% (1.5%)
Pre- to Post change	−1.1%	−1.3%
Difference-in-differences (adjusted)	0.03% (95% CI: −0.13, 0.19%)	
B. Heart failure		
Pre-HVBP: Mean (SD)	11.0% (1.6%)	10.9% (1.6%)
Post-HVBP: Mean (SD)	11.6% (1.5%)	11.4% (1.6%)
Pre- to Post change	0.6%	0.5%
Difference-in-differences (adjusted)	0.04% (95% CI: −0.08, 0.16)	
C. Pneumonia		
Pre-HVBP: Mean (SD)	11.4% (1.9%)	11.4% (1.9%)
Post-HVBP: Mean (SD)	11.7% (1.8%)	11.6% (1.8%)
Pre- to Post change	0.3%	0.02%
Difference-in-differences (adjusted)	0.11% (95% CI: −0.04, 0.27)	

Sources: Authors’ analysis of Hospital Compare (2009–2016), American Hospital Association Annual Survey (2009), and Final Impact Rules (2009–2016) data

Observed average mortality rates reported for pre-HVBP and post-HVBP periods. Difference in average mortality rate between post- vs. pre-HVBP periods reported for high and low Medicare share hospitals based on linear random effects model regressing mortality rate on post-HVBP indicator; heteroscedasticity-robust standard errors clustered at the hospital level

Difference-in-differences estimates from random effects model reported; heteroscedasticity-robust standard errors clustered at the hospital level; covariates in the model include teaching hospital status, ownership, bed size, year and region

*SD* Standard deviation

*CI* Confidence interval

*p* < 0.05

The validity of our difference-in-differences results rests on the assumption that there were no pre-HVBP trend differences between high and low Medicare share hospitals. Therefore, we compared the pre-HVBP mortality trends between high and low Medicare share hospitals (“parallel trends test”). For HF and pneumonia admission cohorts the pre-HVBP trends were similar among the two hospital groups (Table 3 in Appendix); for the AMI cohort, the difference in change between low vs. high Medicare share hospitals was significant but small and not meaningful, amounting to less than 9% of standard deviation in 30-day mortality in 2009.

Sensitivity analysis limiting the post-period to 3 years (2011–2013 and 2014–2016), instead of the 6 years in the main analysis, showed no significant change in mortality – and in the case of pneumonia cohort using 2014–2016 data, a significant not meaningful change – associated with HVBP (Tables 5 and 6 in Appendix). Also, alternative cutoffs of 50 and 60% in identifying high Medicare bed share hospitals also showed no change in findings, with one exception (Tables 7 and 8 in Appendix). Using a lower threshold (50%) showed a significant increase in mortality for pneumonia admissions; however, the magnitude of this change was small, amounting to 10% of the baseline standard deviation in mortality. An alternative regression model specification including hospital fixed effects also produced similar results (Table 9 in Appendix). Including hospital 30-day readmission rate as a covariate showed no change in mortality associated with HVBP and no association between mortality and readmission rates (Table 10 in Appendix). In models to examine heterogeneity we found significant increase in mortality (of up to 25% of the standard deviation in mortality rate) for low bed size (AMI mortality) and privately owned (HF and pneumonia mortality) hospitals (Table 11 in Appendix).

## Discussion

Our main findings showed that exposure to larger HVBP penalties was not associated with differential change in 30-day mortality for AMI, HF and pneumonia patients across hospitals. These findings were largely reiterated in extensive sensitivity analyses; although in a few cases we found a significant change in mortality associated with HVBP exposure, in all these instances the magnitude of change was small (under 25% of the standard deviation in the mortality measure at baseline). These results are broadly consistent with findings from other studies that used critical access hospitals or hospitals in the state of Maryland as the comparison group [5, 6]. Additionally, we did not find evidence of mortality spillover effects from changes in readmission rates due to the HRRP (i.e., higher mortality rates for targeted conditions). Our study adds to the body of evidence in the literature that has found no significant impact of pay-for-performance programs on patient outcomes [27], but extends existing work in two important ways. First, we utilized the design of the HVBP to identify hospitals that differed in the extent of HVBP incentives. This provided the method to compare IPPS hospitals that were more likely to be affected by the program (larger effective penalty) with IPPS hospitals that were less likely to be impacted by the program (smaller effective penalty), in the post-HVBP period compared to the pre-HVBP period. Previous studies relied on non-IPPS hospitals to serve as a control group for hospitals that were exposed to the program; as noted previously, non-IPPS hospitals were different from IPPS hospitals in ways that potentially limited their comparability in the 30-day mortality experience [5, 6]. Nevertheless, our finding of no meaningful change in mortality following the HVBP using a comparison between hospitals that differed in the effective incentive size under HVBP, is comparable to the results of prior studies using non-IPPS hospitals as controls and, taken together, all the studies reinforce the finding of no HVBP-associated changes in 30-day mortality. Second, while other studies have assessed the mortality outcomes over a relatively short follow-up period (one to two years) after the start of the HVBP, we evaluated the outcomes of the IPPS hospitals over a longer follow-up period (4 years), reducing the risk of missing late effects of the HVBP [5, 6].

There are a number of possible explanations for the lack of evidence of improvement in patient outcomes under the HVBP. First, the financial incentives under the HVBP may have been too small – the maximum penalty was 1% (2013), 1.25% (2014), 1.5% (2015), and 1.75% (2016) – to elicit performance changes from hospitals [28, 29]. Second, the HVBP is spread out over multiple domains – patient experience, process of care, patient outcomes, and efficiency – with the result that the effect of the program may be dispersed and hard to appreciate [1, 5]. Third, even though we have 4 years of follow-up data, bringing about systematic changes in hospital care that can result in improvement in mortality may require even more time. It remains to be seen whether reductions in mortality will require a longer time to materialize. Fourth, it may be that pay-for-performance schemes that give financial rewards or penalties to providers for better performance are not effective enough to impact the outcomes we examined. In fact, prior studies have failed to find any evidence that pay-for-performance schemes have improved patient outcomes [27, 30].

Our study has several limitations. First, although reliance on Medicare revenues may be smaller for the low Medicare share hospitals, these hospitals still have an incentive to improve patient outcomes since part of their revenue comes from Medicare payments. However, the magnitude of this incentive is likely to be substantially smaller in comparison with that for high Medicare share hospitals. Thus, to the extent that incentives influence hospital behavior, efforts to reduce mortality under the HVBP should have been substantially greater in high-Medicare share hospitals. Second, the HVBP program was launched during the time that quality of care was improving [1]. While this secular trend may limit our ability to disentangle the effects of the HVBP from the concurrent changes in hospital quality of care, our use of a difference-in-differences analysis is the approach most likely to achieve this. Third, our models do not account for time-varying factors, such as changes in regional hospital competition and state healthcare policy (e.g., Medicaid policy), that may systematically affect high and low Medicare share hospitals differently, leading to potential bias in our estimates of the change in mortality associated with HVBP.

## Conclusion

In conclusion, our results indicate that HVBP was not associated with differential improvement in mortality outcomes across hospitals that differed in the extent of risk of penalty over the 4 years since the implementation of the program. Policy makers should re-evaluate whether providing monetary incentives to hospitals is an effective mechanism to motivate hospitals to perform better. There is considerable concern that this program may have unintended adverse consequences, particularly for financially vulnerable hospitals, by exacerbating the resource constraints in facilitating interventions to improve patient care; also, they may lead to undesirable gaming responses, such as patient cherry-picking or use of alternative diagnostic codes for non-targeted conditions [31]. Alternatively, CMS should consider working collaboratively – rather than punitively – with hospitals to identify and prioritize quality problems that are most relevant to individual providers, create and support learning systems that focus on collecting data for learning and quality improvement, and provide more financial support for quality improvement efforts at hospitals that lack resources.

## Data Availability

Following are the four sources of data used for this study; all data are publicly available. 1) CMS Hospital Compare. Centers for Medicare & Medicaid Services: Hospital Compare Data Archive. https://data.medicare.gov/data/archives/hospital-compare; 2018. 2) CMS Final Rule Impact File. Centers for Medicare & Medicaid Services: Final Rule Impact File Data. https://www.cms.gov/Medicare/Medicare-Fee-for-Service-Payment/AcuteInpatientPPS/Historical-Impact-Files-for-FY-1994-through-Present.html; 2017. 3) AHA Annual Survey. American Hospital Association: AHA Annual Survey Database. Chicago: www.ahadata.com; 2017. 4) American Community Survey. U.S. Census Bureau: American Community Survey. In., vol. https://factfinder.census.gov/. Washington, DC: U.S. Census Bureau; 2017.

## Appendix

This document contains (a) estimates of all covariates from the regression models, and (b) estimates from regression models of sensitivity analyses.

**Table 3:** Parallel trends test for association of HVBP with 30-day risk adjusted mortality rate for acute myocardial infarction (AMI), heart failure (HF), and pneumonia

	AMI	HF	Pneumonia
High Medicare share hospitals	− 0.084 (0.11)	− 0.100 (0.09)	− 0.074 (0.11)
Post 2010	−0.322* (0.04)	0.125* (0.03)	0.006 (0.05)
High Medicare share hospitals x Post 2010	0.151* (0.07)	0.045 (0.05)	0.028 (0.05)
Teaching hospital	−0.346* (0.16)	− 0.164 (0.12)	0.276 (0.15)
Government non-federal (ref: not-for-profit)	0.346* (0.16)	0.106 (0.11)	0.572* (0.15)
For-profit (ref: not-for-profit)	0.197 (0.12)	−0.135 (0.10)	0.057 (0.12)
Bed size: 100–199 (ref: < 100)	0.431* (0.18)	−0.079 (0.10)	−0.202 (0.13)
Bed size: > = 200 (ref: < 100)	0.462* (0.18)	−0.114 (0.11)	−0.285* (0.13)
Midwest (ref: Northeast)	0.090 (0.13)	0.134 (0.11)	−0.243 (0.12)
South (ref: Northeast)	0.476* (0.16)	0.281* (0.13)	0.098 (0.160)
West (ref: Northeast)	0.182 (0.154)	0.791* (0.13)	0.321* (0.15)
DSH %	1.55* (0.32)	−0.986* (0.20)	0.245 (0.25)
Average daily census	−0.002* (0.0004)	−0.002* (0.0003)	− 0.002* (0.0004)
Poverty rate	0.007 (0.30)	0.064* (0.02)	0.036 (0.02)
Unemployment rate	−0.014 (0.025)	−0.096* (0.02)	− 0.007 (0.22)
N	2900	3740	3762

Sources: Authors’ analysis of Hospital Compare (2009–2010), American Hospital Association Annual Survey (2009), and Final Impact Rules (2009–2010) data

Here only data from 2009 and 2010 was used and change between 2009 and 2010 was compared between high vs. low Medicare share hospitals

Post 2010: Includes year 2010

High Medicare share hospitals: Hospitals with % Medicare inpatient days > = 55%; Low Medicare share hospitals: Hospitals with % Medicare inpatient days < 55%

Estimates from random effects model reported; heteroscedasticity-robust standard errors clustered at the hospital level; model includes year dummies

Standard errors in parenthesis

**p* < 0.05

**Table 4:** Association of HVBP with 30-day risk adjusted mortality rate for acute myocardial infarction (AMI), heart failure (HF), and pneumonia– full regression results

	AMI	HF	Pneumonia
High Medicare share hospitals	−0.056 (0.10)	− 0.113 (0.08)	−0.200* (0.10)
Post-HVBP	−2.057* (0.11)	0.467* (0.09)	−0.105 (0.08)
High Medicare share hospitals x Post-HVBP	**0.031** (0.08)	**0.039** (0.06)	**0.114** (0.08)
Teaching hospital	−0.143 (0.12)	− 0.087 (0.11)	0.019 (0.13)
Government non-federal (ref: not-for-profit)	0.358* (0.10)	0.098 (0.08)	0.518* (0.12)
For-profit (ref: not-for-profit)	0.249* (0.09)	−0.202* (0.08)	0.126 (0.10)
Bed size: 100–199 (ref: < 100)	0.304* (0.12)	−0.033 (0.08)	−0.121 (0.11)
Bed size: > = 200 (ref: < 100)	0.306* (0.12)	−0.066 (0.09)	−0.011 (0.12)
Midwest (ref: Northeast)	0.061 (0.09)	0.229* (0.09)	0.017 (0.10)
South (ref: Northeast)	0.442* (0.11)	0.517* (0.10)	0.469* (0.12)
West (ref: Northeast)	0.056 (0.10)	0.543* (0.10)	0.238* (0.12)
DSH %	0.869* (0.23)	−0.542* (0.17)	−0.131 (0.20)
Average daily census	−0.002* (0.0002)	−0.002* (0.0003)	− 0.002* (0.0003)
Poverty rate	0.011 (0.018)	0.006 (0.02)	−0.005 (0.02)
Unemployment rate	0.007 (0.023)	−0.035 (0.02)	−0.008 (0.02)
N	11,600	14,960	15,048

Sources: Authors’ analysis of Hospital Compare (2009–2016), American Hospital Association Annual Survey (2009), and Final Impact Rules (2009–2016) data

High Medicare share hospitals: Hospitals with Medicare share inpatient days > = 55%; Low Medicare share hospitals: Hospitals with Medicare share inpatient days < 55%

Post-HVBP refers to the period 2011–2016

Estimates from random effects model reported; heteroscedasticity-robust standard errors clustered at the hospital level; model includes year dummies

The difference-in-differences estimates are indicated in bold

**p* < 0.05

^a^Standard errors in parenthesis

**Table 5:** Association of HVBP with 30-day risk adjusted mortality rate for AMI, HF, and pneumonia using 2011–2013 as post period

	AMI	HF	Pneumonia
High Medicare share hospitals	−0.040 (0.10)	− 0.103 (0.08)	− 0.122 (0.10)
Post-HVBP	−1.212* (0.08)	0.539* (0.06)	0.360 (0.07)
High Medicare share hospitals x Post-HVBP	**0.093** (0.08)	**0.053** (0.06)	**0.034** (0.08)
N	7250	9350	9405

Note: Only selected estimates reported

Sources: Authors’ analysis of Hospital Compare (2009–2013), American Hospital Association Annual Survey (2009), and Final Impact Rules (2009–2013) data

High Medicare share hospitals: Hospitals with Medicare share inpatient days > = 55%; Low Medicare share hospitals: Hospitals with Medicare share inpatient days < 55%

Post-HVBP refers to the period 2011–2013

Estimates from random effects model reported; heteroscedasticity-robust standard errors clustered at the hospital level; model includes year dummies

Standard errors in parenthesis

**p* < 0.05.

**Table 6:** Association of HVBP with 30-day risk adjusted mortality rate for AMI, HF, and pneumonia using 2014–2016 as post period

	AMI	HF	Pneumonia
High Medicare share hospitals	−0.08 0.099	− 0.039 0.08	−0.15 0.1
Post-HVBP	−2.042* 0.06	0.610* 0.04	−0.087 0.05
High Medicare share hospitals x Post-HVBP	**−0.027** -0.1	**0.034** 0.08	**0.201*** 0.09
N	7250	9350	9405

Note: Only selected estimates reported

Sources: Authors’ analysis of Hospital Compare (2009–2016), American Hospital Association Annual Survey (2009), and Final Impact Rules (2009–2016) data

High Medicare share hospitals: Hospitals with Medicare share inpatient days > = 55%; Low Medicare share hospitals: Hospitals with Medicare share inpatient days < 55%

Post-HVBP refers to the period 2014–2016

Estimates from random effects model reported; heteroscedasticity-robust standard errors clustered at the hospital level; model includes year dummies

* *p* < 0.05.

^a^Standard errors in parenthesis

**Table 7:** Association of HVBP with 30-day risk adjusted mortality rate for AMI, HF, and pneumonia using alternative cut-off point 50% to classify high and low Medicare share hospitals

	AMI	HF	Pneumonia
High Medicare share hospitals	−0.181* (0.09)	− 0.175* (0.08)	− 0.354* (0.10)
Post-HVBP	−2.058* (0.11)	0.437* (0.10)	−0.149 (0.11)
High Medicare share hospitals x Post-HVBP	**0.047** (0.07)	**0.101** (0.08)	**0.194*** (0.07)
Teaching hospital	−0.174 (0.12)	−0.102 (0.11)	− 0.021 (0.13)
Government non-federal (ref: not-for-profit)	0.358* (0.10)	0.095 (0.08)	0.515* (0.12)
For-profit (ref: not-for-profit)	0.248* (0.08)	−0.203* (0.08)	0.126 (0.10)
Bed size: 100–199 (ref: < 100)	0.285* (0.12)	−0.037 (0.08)	−0.133 (0.11)
Bed size: > = 200 (ref: < 100)	0.289* (0.12)	−0.069 (0.09)	−0.024 (0.12)
Midwest (ref: Northeast)	0.067 (0.09)	0.231* (0.09)	0.023 (0.10)
South (ref: Northeast)	0.449* (0.11)	0.517* (0.10)	0.472* (0.12)
West (ref: Northeast)	0.014 (0.10)	0.526* (0.10)	0.189* (0.12)
DSH %	0.800* (0.23)	−0.559* (0.17)	−0.185 (0.21)
Average daily census	−0.002* (0.0002)	−0.002* (0.0003)	− 0.002* (0.0003)
Poverty rate	0.011 (0.018)	0.006 (0.02)	−0.003 (0.02)
Unemployment rate	0.010 (0.023)	−0.034 (0.02)	−0.006 (0.02)
N	11,600	14,960	15,048

Sources: Authors’ analysis of Hospital Compare (2009–2016), American Hospital Association Annual Survey (2009), and Final Impact Rules (2009–2016) data

High Medicare share hospitals: Hospitals with Medicare share inpatient days > = 50%; Low Medicare share hospitals: Hospitals with Medicare share inpatient days < 50%

Post-HVBP refers to the period 2011–2016

Estimates from random effects model reported; heteroscedasticity-robust standard errors clustered at the hospital level; model includes year dummies

The difference-in-differences estimates are indicated in bold

Standard errors in parenthesis

**p* < 0.05

**Table 8:** Association of HVBP with 30-day risk adjusted mortality rate for AMI, HF, and pneumonia using alternative cut-off point 60% to classify high and low Medicare share hospitals

	AMI	HF	Pneumonia
High Medicare share hospitals	−0.120* (0.13)	− 0.142 (0.10)	−0.249* (0.13)
Post-HVBP	−2.052* (0.11)	0.484* (0.09)	−0.083 (0.11)
High Medicare share hospitals x Post-HVBP	**0.033** (0.11)	**−0.054** (0.08)	**0.063** (0.10)
Teaching hospital	−0.143 (0.12)	−0.083 (0.11)	0.027 (0.12)
Government non-federal (ref: not-for-profit)	0.358* (0.10)	0.099 (0.08)	0.518* (0.12)
For-profit (ref: not-for-profit)	0.252* (0.08)	−0.200* (0.08)	0.130 (0.10)
Bed size: 100–199 (ref: < 100)	0.299* (0.12)	0.081 (0.08)	−0.127 (0.11)
Bed size: > = 200 (ref: < 100)	0.299* (0.12)	−0.073 (0.09)	− 0.018 (0.12)
Midwest (ref: Northeast)	0.061 (0.09)	0.230* (0.09)	0.016 (0.10)
South (ref: Northeast)	0.442* (0.11)	0.520* (0.10)	0.471* (0.12)
West (ref: Northeast)	0.053 (0.10)	0.544* (0.10)	0.245* (0.12)
DSH %	0.861* (0.22)	−0.551* (0.17)	−0.132 (0.20)
Average daily census	−0.002* (0.0003)	− 0.002* (0.0003)	− 0.002* (0.0003)
Poverty rate	0.010 (0.018)	0.005 (0.02)	−0.006 (0.02)
Unemployment rate	0.007 (0.023)	−0.035 (0.02)	−0.008 (0.02)
N	11,600	14,960	15,048

Sources: Authors’ analysis of Hospital Compare (2009–2016), American Hospital Association Annual Survey (2009), and Final Impact Rules (2009–2016) data

High Medicare share hospitals: Hospitals with Medicare share inpatient days > = 60%; Low Medicare share hospitals: Hospitals with Medicare share inpatient days < 60%

Post-HVBP refers to the period 2011–2016

Estimates from random effects model reported; heteroscedasticity-robust standard errors clustered at the hospital level; model includes year dummies

The difference-in-differences estimates are indicated in bold

Standard errors in parenthesis

**p* < 0.05

**Table 9:** Association of HVBP with 30-day risk adjusted mortality rate for AMI, HF, and pneumonia using hospital fixed effects regression specification

	AMI	HF	Pneumonia
Post-HVBP	−2.059* (0.054)	0.607* (0.04)	−0.064 (0.05)
High Medicare share hospitals x Post-HVBP	**0.038** (0.08)	**0.047** (0.06)	**0.120*** (0.08)
N	11,600	14,960	15,048

Sources: Authors’ analysis of Hospital Compare (2009–2016), American Hospital Association Annual Survey (2009), and Final Impact Rules (2009–2016) data

High Medicare share hospitals: Hospitals with Medicare share inpatient days > = 55%; Low Medicare share hospitals: Hospitals with Medicare share inpatient days < 55%

Post-HVBP refers to the period 2011–2016

Estimates from hospital fixed effects model reported; heteroscedasticity-robust standard errors clustered at the hospital level; model includes year dummies

The difference-in-differences estimates are indicated in bold

Standard errors in parenthesis

**p* < 0.05

**Table 10:** Association of HVBP with 30-day risk adjusted mortality rate for AMI, HF and pneumonia including 30-day readmission rate as covariate

	AMI	HF	Pneumonia
30-day readmission rate	0.006 (0.02)	0.011 (0.01)	− 0.078 (0.10)
High Medicare share hospitals	−0.058 (0.10)	− 0.117 (0.08)	− 0.218* (0.10)
Post-HVBP	− 2.039* (0.12)	0.488* (0.09)	−0.010 (0.11)
High Medicare share hospitals x Post-HVBP	**0.031** (0.08)	**0.040** (0.06)	**0.115** (0.08)
Teaching hospital	−0.144 (0.12)	−0.088 (0.11)	− 0.003 (0.13)
Government non-federal (ref: not-for-profit)	0.357* (0.10)	0.097 (0.08)	0.510* (0.12)
For-profit (ref: not-for-profit)	0.247* (0.09)	−0.206* (0.08)	0.103 (0.10)
Bed size: 100–199 (ref: < 100)	0.303* (0.12)	−0.033 (0.08)	−0.134 (0.11)
Bed size: > = 200 (ref: < 100)	0.306* (0.12)	−0.064 (0.09)	−0.018 (0.12)
Midwest (ref: Northeast)	0.064 (0.09)	0.238* (0.09)	0.042 (0.10)
South (ref: Northeast)	0.446* (0.11)	0.527* (0.10)	0.511* (0.12)
West (ref: Northeast)	0.062 (0.10)	0.560* (0.10)	0.310* (0.12)
DSH %	0.862* (0.23)	−0.583* (0.17)	−0.201 (0.20)
Average daily census	−0.002* (0.0002)	−0.002* (0.0003)	− 0.002* (0.0003)
Poverty rate	0.011 (0.018)	0.006 (0.02)	−0.002 (0.02)
Unemployment rate	0.007 (0.023)	−0.036 (0.02)	−0.009 (0.02)
N	11,600	14,960	15,048

Sources: Authors’ analysis of Hospital Compare (2009–2016), American Hospital Association Annual Survey (2009), and Final Impact Rules (2009–2016) data

High Medicare share hospitals: Hospitals with Medicare share inpatient days > = 55%; Low Medicare share hospitals: Hospitals with Medicare share inpatient days < 55%

Post-HVBP refers to the period 2011–2016

Estimates from random effects model reported; heteroscedasticity-robust standard errors clustered at the hospital level; model includes year dummies

**p* < 0.05

^a^Standard errors in parenthesis

**Table 11:** Association of HVBP with 30-day risk adjusted mortality rate for AMI, HF, and pneumonia: Heterogeneity in covariates

	AMI	HF	Pneumonia
1. Regression model with heterogeneity in hospital teaching status			
Not teaching hospitals	0.087	0.071	0.066
	0.08	0.066	0.079
Teaching hospitals	0.189	−0.381	−0.585
	0.52	0.361	0.53
2. Regression model with heterogeneity in hospital ownership status			
Not for-profit	−0.014	−0.082	− 0.008
	0.10	0.08	0.09
Government	0.412	0.165	0.256
	0.26	0.18	0.22
For-profit	0.054	0.397*	0.438*
	0.19	0.13	0.17
3. Regression model with heterogeneity in hospital bed size capacity			
100 or fewer beds	0.556*	−0.001	0.068
	0.26	0.13	0.169
100 to 199 beds	0.035	−0.07	0.015
	0.15	0.12	0.15
200 or more beds	−0.045	0.161	0.274
	0.10	0.09	0.11

Note: Only the estimates of heterogeneity in interaction effects reported

Sources: Authors’ analysis of Hospital Compare (2009–2016), American Hospital Association Annual Survey (2009), and Final Impact Rules (2009–2016) data

High Medicare share hospitals: Hospitals with Medicare share inpatient days > = 55%; Low Medicare share hospitals: Hospitals with Medicare share inpatient days < 55%

Post-HVBP refers to the period 2011–2016

Estimates from random effects model reported; heteroscedasticity-robust standard errors clustered at the hospital level; model includes year dummies

**p* < 0.05

^a^Standard errors in parenthesis

## Abbreviations

ACA: Affordable Care Act
AMI: Acute myocardial infarction
CMS: Centers for Medicare and Medicaid Services
HBVP: Hospital Value Based Purchasing Program
HF: Heart Failure
HRRP: Hospital Readmissions Reduction Program
IPPS: Inpatient Prospective Payment System
